# Body Composition, Energy Availability, Risk of Eating Disorder, and Sport Nutrition Knowledge in Young Athletes

**DOI:** 10.3390/nu15061502

**Published:** 2023-03-21

**Authors:** Meghan K. Magee, Margaret T. Jones, Jennifer B. Fields, Julie Kresta, Chinguun Khurelbaatar, Christopher Dodge, Brandon Merfeld, Abby Ambrosius, Makenna Carpenter, Andrew R. Jagim

**Affiliations:** 1 Frank Pettrone Center for Sports Performance, George Mason University, Fairfax, VA 22030, USA; 2 Kinesiology, School of Sport Recreation and Tourism Management, George Mason University, Manassas, VA 22030, USA; 3 Sport, Recreation, and Tourism Management, George Mason University, Fairfax, VA 22030, USA; 4 Exercise Science and Athletic Training, Springfield College, Springfield, MA 01109, USA; 5 Exercise Physiology and Human Performance, Texas A&M University-Central Texas, Killeen, TX 76549, USA; 6 Exercise & Sport Science, University of Wisconsin–La Crosse, La Crosse, WI 54601, USA; 7 Sports Medicine, Mayo Clinic Health System, La Crosse, WI 54601, USA

**Keywords:** eating disorder, disordered eating, energy availability, athletes, nutrition knowledge

## Abstract

Young athletes may be at risk for low energy availability (LEA) or dietary habits that are indicative of eating disorders. Thus, the purpose of the current study was to investigate the prevalence of LEA among high school athletes and examine those at risk for eating disorders. A secondary aim was to examine relationships between sport nutrition knowledge, body composition, and LEA. Methods: 94 male (*n* = 42) and female (*n* = 52) mean ± SD age: 18.09 ± 2.44 y; height: 172.6 ± 9.8 cm; body mass: 68.7 ± 14.5 kg; BMI: 22.91 ± 3.3 kg·m^−2^) athletes completed a body composition assessment and electronic versions of the abridged sports nutrition knowledge questionnaire (ASNK-Q), brief eating disorder in athletes questionnaire (BEDA-Q), and the low energy availability for females questionnaire (LEAF-Q; females only). Results: 52.1% of female athletes were classified as being at risk for LEA. Moderate inverse relationships existed for computed LEAF-Q scores and BMI (r = −0.394; *p* < 0.01). A total of 42.9% of males (*n* = 18) and 68.6% of females (*n* = 35) were at risk for eating disorders, with females being at greater risk (*p* < 0.01). Body fat percentage was a predictor (β = −0.095; *p* = −0.01) for eating disorder risk status. For every 1 unit increase in body fat percentage, athletes were 0.909 (95% CI: 0.845–0.977) times less likely to be classified as at risk for an eating disorder. Male (46.5 ± 13.9) and female (46.9 ± 11.4) athletes scored poorly on the ASNK-Q, with no differences between sex (*p* = 0.895). Conclusions: Female athletes were at a greater risk for eating disorders. No relationships existed between sport nutrition knowledge and %BF. Female athletes with a higher %BF had a lower risk for an eating disorder and risk for LEA.

## 1. Introduction

Previous research has indicated that young athletes often fail to meet recommended dietary guidelines for their sport and activity level [1,2,3,4,5,6,7,8]. In turn, this can increase the likelihood of the athlete presenting with low energy availability (LEA) and relative energy deficiency in sport (RED-S), which subsequently poses a risk for a multitude of long-term negative health and performance implications [9,10]. Estimates regarding the prevalence of LEA among collegiate and young-adult athletes range between 20–60%, depending upon the specific sport, level of competition, and phase of season [6,7,11,12,13,14,15]. Moreover, there are several contributing factors, such as body image, nutrition knowledge, and participation in weight class sports, that have been posited to play a role in the development of these observed energy deficiencies. While important information can be derived from the direct assessment of energy availability among athletes, the measurement of energy intake, energy expenditure, and body composition can be costly, labor intensive, and time consuming [16]. As such, survey tools, such as the low energy availability in females questionnaire (LEAF-Q) and the RED-S clinical assessment tool (RED-S CAT) [17], have been utilized as an efficient way to screen those at risk for LEA and RED-S [18].

Dietary habits that are indicative of disordered eating or clinical eating disorders may also predispose athletes to exhibiting LEA, as the prevalence of eating disorders has been found to be higher in athletes compared to the general population [19]. Moreover, disordered eating patterns underpin a key tenet of LEA, in that the resulting low energy intake is likely insufficient to meet the high energy output of the athlete [10]. Recent evidence suggests that eating behaviors among athletes are best viewed on a spectrum, with dietary behaviors representative of optimized nutrition on one end and a clinical eating disorder on the other [20]. The prevalence of disordered eating and eating disorders among adult athletes ranges from 0–19% and 4–45% for men and women, respectively; yet the numbers may actually be higher due to underreporting and the secretive nature of the disease [19,20,21]. As such, early screening and detection are imperative to ensure that athletes are offered early treatment options and behavioral health counseling if needed. However, screening for eating disorders in athletes may differ from screening the general population, due to the addition of their training factors that may overlap with some more traditional behaviors observed with eating disorders. These behaviors can include dietary restrictions or excessive exercise, often utilized in an effort to modify body weight and/or composition. For the general population, these may be problematic; however, they may be normal, or perceived as such, for some athletes as these practices may be engrained in the culture of the sport [22]. Therefore, specialized screening tools targeting athletes are imperative in order to accurately detect eating disorders or identify those at risk for developing one in the future. Recently, a brief eating disorder in athletes questionnaire has been developed as a screening tool [23]. Deployment of these tools can be a useful strategy for evaluating the prevalence of eating disorder behaviors across different sport types, and can play a pivotal role in early detection.

Another area of concern, which likely influence dietary habits and the subsequent risk of LEA and disordered eating, is the lack of understanding regarding the specific nutritional requirements of the sport [5,10,24,25]. This, in addition to a lack of access to credible sources of information can be problematic for athletes in regard to optimized fueling strategies for performance [26,27,28]. Further, athletes often face an array of logistical barriers that can make it difficult to meet the nutritional recommendations for their sport. Specifically, at the elite and collegiate level, athletes have indicated that a lack of time for food preparation, financial limitations, inadequate cooking skills, lack of knowledge, and difficulty with living arrangements are barriers to healthy eating [26,29]. Younger athletes (e.g., high school level) may be faced with similar barriers to nutrition, specifically a lack of knowledge and time [30]; however, they may have the support of a parent/guardian at home to help with food expenses and cooking, which likely differs from a collegiate athlete lifestyle. Regardless, it is clear that a large majority of athletes across multiple levels of competition lack an appropriate understanding of specific nutritional strategies to optimize performance and health. In turn, this may increase their risk for disordered eating behaviors, the likelihood of under-fueling, and LEA.

Previous studies have linked sport nutrition knowledge to healthy eating behaviors. In a study examining high school athletes, researchers used a two-year nutritional education intervention to observe the impact of a nutrition education intervention on dietary beliefs and behaviors. Following the educational intervention, there were significant improvements in not only overall sport nutrition knowledge, but improvements in healthy eating behaviors, such as not skipping meals, which may lead to a low energy intake. The authors also noticed an increase in motivation to eat for performance, which included factors such as consuming fuel and fluids one hour prior to practice or competition [31]. This further supports the importance of adequate nutrition knowledge for young athletes, and the role nutrition education interventions can play in reducing risks for LEA and other nutrition-based consequences of inadequate dietary habits in young athletes.

Through a better understanding of the knowledge gaps among young athletes relative to nutrition knowledge, in conjunction with evaluating the prevalence of LEA and the risk of eating disorders, a more targeted approach can be implemented to educate young athletes on healthy dietary habits and fueling strategies for optimizing athletic performance. Therefore, the purpose of the current study was to investigate the prevalence of LEA among young female athletes and the risk of eating disorders for both female and male athletes. A secondary aim was to examine sport nutrition knowledge and potential relationships between body composition and risk for eating disorder.

## 2. Materials and Methods

### 2.1. Study Design

Prior to the start of the 2021–2022 athletic season, high school, and collegiate athletes from a variety of sports (i.e., wrestling, soccer, volleyball, basketball, track and field, football and tennis) were recruited to participate. Athletes reported to the laboratory for a single day of testing, which included a body composition assessment and the completion of multiple electronic questionnaires to assess the risk for low energy availability (females only), eating disorder risk, and nutrition knowledge.

### 2.2. Subjects

A total of 94 young male (*n* = 42) and female (*n* = 52) ([mean ± SD] age: 18.09 ± 2.44 y; height: 172.6 ± 9.8 cm; body mass: 68.7 ± 14.5 kg; BMI: 22.91 ± 3.3 kg·m^−2^) athletes participated in the current study. Athletes from soccer (*n* = 28), football (*n* = 12), track/cross-country (*n* = 10), wrestling (*n* = 9), baseball (*n* = 7), volleyball (*n* = 5), gymnastics (*n* = 5), basketball (*n* = 4), weight/power lifting (*n* = 4), dance (*n* = 1), softball (*n* = 2), tennis (*n* = 1), CrossFit (*n* = 1), skiing (*n* = 1), and hockey (*n* = 1) were represented. Forty of the athletes (43.9%) indicated that they participate in two sports, 19 (20.8%) in three sports, and 3 (3.3%) in four sports. All participants provided informed consent or assent (for those less than 18 years of age) approved by the University’s Institutional Review Board and all study procedures were conducted according to the Declaration of Helsinki and Human Subjects Research Guidelines. Parental or guardian consent was provided for participants younger than 18 years of age.

### 2.3. Procedures

#### 2.3.1. Body Composition

Height and weight were initially recorded using a self-calibrating physician’s scale and stadiometer and were later used to determine body mass index (BMI). Participants completed a body composition assessment via air displacement plethysmography (ADP) to determine percent body fat (%BF), fat mass, and fat free mass (FFM) using the Brozek equation [32]. Participants were instructed to report to the laboratory in a fasted (>12 h) state and having refrained from any strenuous activity (>24 h) prior to testing. Participants wore spandex with a lycra swim cap and removed all jewelry prior to body composition testing to reduce excess air displacement. Thoracic gas volume was predicted using manufacturer settings. Test to test reliability of body composition assessment using ADP within our lab has yielded high reliability for body mass (r = 0.999), body fat percent (r = 0.994), and fat-free mass (r = 0.998) in athletic populations.

#### 2.3.2. Questionnaires

Three separate questionnaires were deployed electronically using an online survey distribution platform (Qualtrics, Provo, UT, USA).

##### Eating Disorder Risk

To assess eating disorder risk, athletes completed an electronic version of the brief eating disorder in athletes questionnaire (BEDA-Q) [23]. The questionnaire consisted of 9 questions, adopted from the Eating Disorder Inventory (EDI). The scores were computed by summing the item scores. Positive scores were weighted as follows: 3 = always, 2 = usually, 1 = often, 0 = sometimes, 0 = rarely, 0 = never, and reverse-scored items were weighted in the opposite manner. A weighted summed score > 0.27 was considered to be at risk for an eating disorder. Previous research has indicated the BEDA-Q to exhibit good sensitivity of 82.1% (95% CI, 76.6–87.6) and specificity of 84.6% (95% CI, 79.4–89.8). Further, the BEDA-Q has showed acceptable levels of accuracy in distinguishing athletes with and without an eating disorder with a receiver operating characteristics area of 0.86 (95% confidence interval (CI), 0.78–0.93) [23].

#### 2.3.3. Sport Nutrition Knowledge

The abridged sports nutrition knowledge questionnaire (ASNK-Q) [33,34] was used to assess nutrition knowledge with a focus on athletic performance. This questionnaire included 35 total questions designed to assess general (*n* = 11) and sport-specific nutritional recommendations (*n* = 24). The ANSK-Q has been shown to exhibit high construct validity (*p* < 0.001) with good test-to-test concordance (r = 0.80; *p* < 0.001) among young athletes. The submitted responses were instantly scored upon submission to yield an overall composite score and interpreted as “poor” knowledge (0–49%), “average” knowledge (50–65%), “good” knowledge (66–75%), and “excellent” knowledge (75–100%) based on previously published methods [34].

##### Low Energy Availability

The low energy availability for females questionnaire (LEAF-Q) [18] was used to screen female athletes for the risk of low energy availability. The LEAF-Q consists of 25 questions centered on prior injury history, gastrointestinal symptoms, menstrual cycle patterns, and contraceptives, all of which pertain to various components surrounding low energy availability in females. The questionnaire is scored with a value >8 indicative of the athlete likely presenting on the spectrum of having low energy availability. The LEAF-Q has been shown to have an acceptable degree of sensitivity (78%) and specificity (90%) with a Cronbach’s alpha > 0.71 among women athletes [18].

### 2.4. Statistical Analysis

An independent samples t-test was run to evaluate the difference in risk status for low energy availability and eating disorder between males and females and by school status (high school versus college) (*p* < 0.05). Binary logistic regression was used to predict the risk of eating disorder. Spearman rank-order correlation coefficients examined relationships between ASNK-Q, BEDA-Q, and LEAF-Q (females only) scores and body composition parameters. Correlation coefficients were calculated to assess relationships and were interpreted as: very weak: <0.20, weak: 0.20–0.39, moderate: 0.40–0.59, strong: 0.60–0.79, and very strong: >0.80 [35]. All data were analyzed using the Statistical Package for the Social Sciences (IBM SPSS Statistics for Windows, Version 26.0: IBM Corp. Armonk, NY, USA).

## 3. Results

Athlete characteristics are included in Table 1. Based upon results from the LEAF-Q, 52.1% of female athletes were classified as being at risk for LEA. A higher percentage of high school female athletes (53.8%) were at risk for LEA compared to collegiate female athletes (48.5%), albeit not statistically significant (*p* = 0.107). Moderate inverse relationships were observed for computed LEAF-Q scores and BMI (r = −0.394; *p* < 0.01) and body fat percentage (r = −0.411; *p* < 0.01).

According to the weighted score on the BEDA-Q, 42.9% of males (*n* = 18) and 68.6% of females (*n* = 35) were at risk for eating disorders with females at a higher risk (*p* < 0.01). A summary of responses to the individual questions from the BEDA-Q can be found in Table 2.

The overall model including ASNKQ score, BMI, and body fat percentage accurately predicted those at risk of an eating disorder 84.6% of the time. Body fat percentage was a significant predictor (β = −0.095; *p* = −0.01) for eating disorder risk status, in that for every 1 unit increase in an athlete’s body fat percentage, they were 0.909 (95% CI: 0.845–0.977) times less likely to be classified as being classified at risk for an eating disorder.

Male (46.5 ± 13.9) and female (46.9 ± 11.4) athletes scored poorly on the ASNK-Q with no differences between sex (*p* = 0.895). Both high school (44.9 ± 10.7) and collegiate (48.9 ± 14.0) athletes scored poorly on the ASNK-Q, with no differences between school level (*p* = 0.103) as summarized in Table 3. There was a moderate association between age and ASNKQ scores (r = 0.317) in male and female athletes (r = 0.341). No other relationship between ASNKQ scores and body composition parameters or computed scores for the BEDA-Q and LEAF-Q were observed (*p* > 0.05).

## 4. Discussion

The primary aim of the current study was to assess the prevalence of LEA and the risk of eating disorder among a cohort of young athletes. Main findings were that 52.1% of the female athletes in the current study were identified as being at risk for LEA. Further, approximately half of the athletes were classified to be at risk for an eating disorder, with a higher percentage of female athletes considered to be at risk than male athletes. Body fat percentage was not associated with risk factors for eating disorders in male athletes. However, female athletes with a higher %BF had a lower risk for eating disorder and LEA.

The prevalence of LEA among the female athletes observed in the current study is slightly higher than previously reported in collegiate and young athletes, particularly those who participate in endurance-based sports, weight-sensitive, and aesthetic sports, whom often have a higher prevalence of LEA [6,10,11,13]. The diverse representation of female athletes from various sports may have contributed to the higher prevalence of LEA observed. Additionally, the younger ages of the participants may have also contributed to the higher prevalence of LEA, as much of the available data has been reported in collegiate, elite, and professional athletes, rather than high school athletes. It is also possible that the LEAF-Q used to identify those with LEA in the current study may overestimate the percentage of athletes who would be classified as having LEA through use of direct measures of energy intake and exercise energy expenditure, as previously noted [7]. However, previous research has revealed that the LEAF-Q is able to accurately identify athletes at risk for LEA [18], with results of the survey tool being associated with markers of low energy available and relative energy deficiency in sport (RED-S) [36].

It has been suggested that LEA and energy deficiency may be underpinned by underlying disordered eating patterns displayed by athletes, or by eating behaviors more representative of an eating disorder that would meet the clinical criteria. Therefore, screening athletes for eating disorder risk may be an effective strategy to identify those individuals who may be at risk for both an eating disorder and, subsequently, LEA. If risk is identified, the athlete should be provided with the necessary resources and treatment plan as determined by members of the sports medicine team. In the current study, 69% of females and 43% of the male athletes were classified as being at risk for eating disorders, which far exceeds the prevalence of eating disorders at 0 to 19% for male and 4 to 45% for female athletes, previously reported [19,20,21]. However, it is worth noting that the survey instrument utilized in the current study is meant to serve as a screening tool rather than clinically accepted diagnostic criteria for diagnosing athletes with an eating disorder. Previous research has found that eating disorders are more common in female athletes compared to male athletes, which is in alignment with findings from the current study [20]. Similar to LEA, weight sensitive, aesthetic, and endurance-based sports tend to have a higher prevalence of eating disorders compared to other sport types. It is difficult to discern primary causality in eating disorder cases, as there are likely several contributing factors (psychological etiologies, societal pressures, sport culture, body image dissatisfaction, etc.) that predispose certain individuals for being at a greater risk of developing an eating disorder [10,20,37,38,39], and none of which were assessed in the current study.

It is important to evaluate additional risk factors that may be associated with the risk of disordered eating patterns. For example, in the current study, %BF was found to be a predictor of risk for eating disorders. Specifically, athletes with a higher %BF were less likely to be classified as being at risk for an eating disorder, suggesting that a certain degree of leanness may indicate a risk for eating disorders. Previous research has indicated that eating disorders appear to be more prevalent in weight-sensitive sports, endurance sports, and physique athletes [10,20], all of which often emphasize low BF%. Additionally, female athletes tend to be at a greater risk for eating disorders when compared to their male counterparts [9,20,40]. An additional health consequence for females with eating disorders or restricted dietary intake is the female athlete triad, which includes low energy availability, menstrual dysfunction, and low bone mineral density. Such physiological changes in young adults could have long term effects on their overall health and athletic performance, supporting the importance of early detection.

While the underlying reasons for an athlete presenting an eating disorder are likely different from those of an athlete who fails to meet the nutritional requirements of their sport (i.e., disordered eating), the outcomes may be similar in nature, yet the extent of health and performance perturbations likely fall on a spectrum, subsequently influenced by the magnitude and duration of energy deficiency [10]. While not assessed in the current study, body image dissatisfaction has been found to be associated with eating disorder risk in both college-aged men and women [10,41,42,43,44] and is often used as part of the screening and diagnostic process [20,22,45].

A secondary aim was to examine sport nutrition knowledge and potential relationships between body composition and risk for eating disorder. Results from the current study indicate that young athletes have ‘poor’ sport nutrition knowledge, as both high school and collegiate athletes scored poorly on the ASNK-Q. Additionally, there were no differences observed between sexes or those at risk versus those not at risk for an eating disorder. It is not uncommon for athletes to struggle with meeting the dietary requirements for their respective sport and activity level, as this has been consistently reported within the literature across multiple levels of competition and sport types [2,4,5,46,47]. Specifically, energy and carbohydrate deficiencies appear to be the most observed deficiencies among athletes [2,4,5,46,47]. In the current study, athletes answered a lower percentage of questions correctly on the sport nutrition-specific section of the questionnaire compared to the general nutrition knowledge section, thereby indicating that the application of nutrition knowledge within the context of sports performance should be an area of focus for athletes. There were no relationships observed between sport nutrition knowledge and %BF in male and female athletes. The lack of sport nutrition knowledge in young athletes could also be related to the knowledge and information they are receiving from their coaches [48]. Studies have examined the sport nutrition knowledge of various professionals involved with a sport team and found that only 35.9% of coaches and 9% of athletes had sufficient nutritional knowledge [25,49]. Therefore, the focus on nutritional education programs could also extend to the coaches and other individuals working with the athletes regularly, which may be an area for future research [49].

There appears to be a positive relationship between nutritional knowledge and dietary intake and healthy dietary behaviors, as previous studies have shown that athletes with higher levels of knowledge are more likely to have higher intakes of overall energy, carbohydrates, fiber, and calcium [50]. Nutrition knowledge has also been associated with increased protein intake in athletes as well as greater consumption of fruits and vegetables, along with a lower intake of fats and sweetened drinks [50]. Therefore, with an increased focus on the importance of nutrition in sport, young athletes may be less likely to develop unhealthy or disordered eating patterns that could potentially lead to complications, such as eating disorders or LEA.

A limitation of the current study was the unequal distribution of athletes across each sport type, which prevented any analysis on the risk of eating disorder by sport type. The lack of follow-up surveillance and diagnostic confirmation regarding the likelihood of a positive screening from the BEDA-Q to accurately identify an athlete with an ED is another limitation of the current study. While the BEDA-Q has been found to yield a high degree of specificity and sensitivity regarding its ability to detect athletes with an ED, the lack of more comprehensive clinical assessment precludes any definitive diagnosis among the current cohort. Lastly, the lack of dietary intake data is another limitation as this information would have indicated whether or not poor sport nutrition knowledge or the risk of eating disorder was associated with an inadequate dietary intake and the direct measurement of low energy availability.

## 5. Conclusions

It is recommended that practitioners working with female high school athletes be made aware of the potential impact of body composition on the risk of developing an eating disorder. Developing a protocol to screen those who may be at risk and then refer those who may be identified as at-risk to appropriate registered dietitians and health care professionals are suggested. Further, it is recommended that sport nutrition education interventions be implemented as part of a long-term athlete development program to ensure that athletes are equipped with the knowledge to adopt appropriate fueling strategies.

## Figures and Tables

**Table 1:** Athlete characteristics.

	Age (yrs)	Height (cm)	Body Mass (kg)	Body Mass Index (kg·m^−2^)	Body Fat (%)	Fat-Free Mass (kg)
**Male (*n* = 42)**	17.0 ± 2.4	179.6 ± 8.2	76.9 ± 17.1	23.7 ± 4.2	13.6 ± 6.6	65.7 ± 11.2
**Female (*n* = 52)**	19.0 ± 2.1	166.6 ± 6.6	61.6 ± 5.9	22.2 ± 2.2	21.6 ± 5.0	48.4 ± 3.9

Values are represented as mean ± standard deviation.

**Table 2:** Summary of responses to BEDA-Q.

	Always		Usually		Often		Sometimes		Rarely		Never	
	M *n* (%)	W *n* (%)	M *n* (%)	W *n* (%)	M *n* (%)	W *n* (%)	M *n* (%)	W *n* (%)	M *n* (%)	M *n* (%)	W *n* (%)	M *n* (%)
*I feel extremely guilty after overeating*	3 (7.1)	7 (13.7)	6 (14.3)	14 (27.5)	2 (4.8)	6 (11.8)	10 (23.8)	13 (25.5)	10 (23.8)	7 (13.7)	10 (23.8)	1 (2.0)
*I am preoccupied with the desire to be thinner*	0 (0)	5 (9.8)	4 (9.5)	4 (7.8)	4 (9.5)	7 (13.7)	8 (19.0)	12 (23.5)	11 (26.2)	16 (31.4)	14 (33.3)	4 (7.8)
*I think that my stomach is too big*	1 (2.4)	5 (9.8)	2 (4.8)	6 (11.8)	0 (0)	7 (13.7)	8 (19.0)	12 (23.5)	17 (40.6)	12 (23.5)	13 (31.0)	6 (11.8)
*I feel satisfied with the shape of my body*	6 (14.3)	0 (0)	15 (35.7)	14 (27.5)	10 (23.8)	13 (25.5)	9 (21.4)	11 (21.6)	1 (2.4)	10 (19.6)	1 (2.4)	0 (0)
*My parents have expected excellence of me*	11 (26.2)	14 (27.5)	9 (21.4)	16 (31.4)	9 (21.4)	6 (11.8)	4 (9.5)	7 (13.7)	4 (9.5)	3 (5.9)	4 (9.5)	2 (3.9)
*As a child, I tried very hard to avoid disappointment my parents and teachers*	9 (21.4)	19 (37.3)	11 (26.2)	13 (25.5)	6 (14.3)	8 (15.7)	7 (16.7)	6 (11.8)	6 (14.3)	1 (2.0)	2 (4.8)	1 (2.0)
	Yes		No									
	M *n* (%)	W *n* (%)	M *n* (%)	W *n* (%)								
*Are you trying to lose weight now?*	6 (14.3)	8 (15.7)	35 (83.3)	40 (78.4)								
*Have you tried to lose weight?*	21 (50.0)	29 (56.9)	20 (47.6)	19 (37.3)								
	1–2		3–5		>5 times							
	M *n* (%)	W *n* (%)	M *n* (%)	W *n* (%)	M *n* (%)	W *n* (%)						
*If yes, how many times have you tried to lose weight?*	29 (69.0)	29 (56.9)	6 (14.3)	8 (15.7)	5 (11.9)	10 (19.6)						
*Have you experienced menstrual dysfunction or amenorrhea (primary or secondary) in the past 6 months?*		7 (13.7)		41 (80.4)								

Values are represented as number (*n*) of responses and percentage of total (%). M = men athletes; W = women athletes.

**Table 3:** Nutrition knowledge scores for the total sample and subgroup analysis.

	(*n*)	Total	% Correct	GNK	% Correct	SNK	% Correct
School Status							
High School	42	15.6 ± 3.7	44.5 ± 10.7	6.15 ± 2.2	55.9 ± 19.9	8.0 ± 2.6	33.3 ± 10.8
College	47	17.5 ± 4.2 *	48.9 ± 13.9	6.42 ± 1.9	58.4 ± 17.1	9.3 ± 2.3	38.8 ± 13.0
Sex							
Male	42	16.7 ± 4.2	46.5 ± 13.9	6.3 ± 1.7	57.6 ± 15.1	9.3 ± 2.6	38.6 ± 10.8
Female	50	16.4 ± 3.9	46.9 ± 11.4	6.3 ± 1.8	56.9 ± 18.0	8.9 ± 2.4	37.4 ± 9.9
EA Status							
LEA	25	16.6 ± 3.1	47.3 ± 8.8	6.4 ± 1.8	58.2 ± 16.0	9.2 ± 1.9	38.5 ± 7.9
Non-LEA	23	16.0 ± 4.7	45.7 ± 13.5	6.1 ± 2.2	55.7 ± 20.2	8.6 ± 2.8	35.9 ± 11.8
Eating Disorder							
At Risk	53	16.1 ± 4.4	45.9 ± 12.5	6.0 ± 1.9	54.7 ± 16.9	9.0 ± 2.6	37.5 ± 11.0
Not At Risk	36	17.0 ± 3.5	48.7 ± 9.9	6.7 ± 1.8	60.9 ± 15.8	9.2 ± 2.3	38.4 ± 9.4

Values are represented as mean ± standard deviation. * Denotes significant difference (*p* < 0.05) between subgroups. EA = energy availability; GNK = general nutrition knowledge; SNK = sports nutrition knowledge.

## Data Availability

Data available upon request.

## References

[1] Clark M., Reed D.B., Crouse S.F., Armstrong R.B. (2003). Pre- and post-season dietary intake, body composition, and performance indices of NCAA division I female soccer players. Int. J. Sport Nutr. Exerc. Metab..

[2] Shriver L.H., Betts N.M., Wollenberg G. (2013). Dietary intakes and eating habits of college athletes: Are female college athletes following the current sports nutrition standards?. J. Am. Coll. Health.

[3] Reed J.L., De Souza M.J., Kindler J.M., Williams N.I. (2014). Nutritional practices associated with low energy availability in Division I female soccer players. J. Sport. Sci..

[4] Jagim A.R., Wright G.A., Kisiolek J., Jones M.T., Oliver J.M. (2016). Position specific changes in body composition, hydration status, and metabolism during preseason training camp and nutritional habits of Division III football players. Open Sport Sci. J..

[5] Jagim A., Zabriskie H., Currier B., Harty P., Stecker R., Kerksick C. (2019). Nutrient Status and perceptions of energy and macronutrient intake in a Group of Collegiate Female Lacrosse Athletes. J. Int. Soc. Sport. Nutr..

[6] Logue D.M., Madigan S.M., Melin A., Delahunt E., Heinen M., Donnell S.M., Corish C.A. (2020). Low Energy Availability in Athletes 2020: An Updated Narrative Review of Prevalence, Risk, Within-Day Energy Balance, Knowledge, and Impact on Sports Performance. Nutrients.

[7] Magee M.K., Lockard B.L., Zabriskie H.A., Schaefer A.Q., Luedke J.A., Erickson J.L., Jones M.T., Jagim A.R. (2020). Prevalence of Low Energy Availability in Collegiate Women Soccer Athletes. J. Funct. Morphol. Kinesiol..

[8] Renard M., Kelly D.T., Cheilleachair N.N., Cathain C.O. (2021). How Does the Dietary Intake of Female Field-Based Team Sport Athletes Compare to Dietary Recommendations for Health and Performance? A Systematic Literature Review. Nutrients.

[9] Areta J.L., Taylor H.L., Koehler K. (2020). Low energy availability: History, definition and evidence of its endocrine, metabolic and physiological effects in prospective studies in females and males. Eur. J. Appl. Physiol..

[10] Jagim A.R., Fields J., Magee M.K., Kerksick C.M., Jones M.T. (2022). Contributing Factors to Low Energy Availability in Female Athletes: A Narrative Review of Energy Availability, Training Demands, Nutrition Barriers, Body Image, and Disordered Eating. Nutrients.

[11] Reed J.L., De Souza M.J., Williams N.I. (2013). Changes in energy availability across the season in Division I female soccer players. J. Sport. Sci..

[12] Sygo J., Coates A.M., Sesbreno E., Mountjoy M.L., Burr J.F. (2018). Prevalence of Indicators of Low Energy Availability in Elite Female Sprinters. Int. J. Sport Nutr. Exerc. Metab..

[13] Melin A.K., Heikura I.A., Tenforde A., Mountjoy M. (2019). Energy Availability in Athletics: Health, Performance, and Physique. Int. J. Sport Nutr. Exerc. Metab..

[14] Dobrowolski H., Wlodarek D. (2020). Low energy availability in group of Polish female soccer players. Rocz. Państwowego Zakładu Hig..

[15] Moss S.L., Randell R.K., Burgess D., Ridley S., Ócairealláin C., Allison R., Rollo I. (2021). Assessment of energy availability and associated risk factors in professional female soccer players. Eur. J. Sport Sci..

[16] Burke L.M., Lundy B., Fahrenholtz I.L., Melin A.K. (2018). Pitfalls of Conducting and Interpreting Estimates of Energy Availability in Free-Living Athletes. Int. J. Sport Nutr. Exerc. Metab..

[17] Mountjoy M., Sundgot-Borgen J., Burke L., Carter S., Constantini N., Lebrun C., Meyer N., Sherman R., Steffen K., Budgett R. (2015). The IOC relative energy deficiency in sport clinical assessment tool (RED-S CAT). Br. J. Sport. Med..

[18] Melin A., Tornberg A.B., Skouby S., Faber J., Ritz C., Sjodin A., Sundgot-Borgen J. (2014). The LEAF questionnaire: A screening tool for the identification of female athletes at risk for the female athlete triad. Br. J. Sport. Med..

[19] Sundgot-Borgen J., Torstveit M.K. (2004). Prevalence of eating disorders in elite athletes is higher than in the general population. Clin. J. Sport Med..

[20] Wells K.R., Jeacocke N.A., Appaneal R., Smith H.D., Vlahovich N., Burke L.M., Hughes D. (2020). The Australian Institute of Sport (AIS) and National Eating Disorders Collaboration (NEDC) position statement on disordered eating in high performance sport. Br. J. Sport. Med..

[21] Reardon C.L., Hainline B., Aron C.M., Baron D., Baum A.L., Bindra A., Budgett R., Campriani N., Castaldelli-Maia J.M., Currie A. (2019). Mental health in elite athletes: International Olympic Committee consensus statement (2019). Br. J. Sport. Med..

[22] Knapp J., Aerni G., Anderson J. (2014). Eating disorders in female athletes: Use of screening tools. Curr. Sport. Med. Rep..

[23] Martinsen M., Holme I., Pensgaard A.M., Torstveit M.K., Sundgot-Borgen J. (2014). The development of the brief eating disorder in athletes questionnaire. Med. Sci. Sport. Exerc..

[24] Heaney S., O’Connor H., Michael S., Gifford J., Naughton G. (2011). Nutrition knowledge in athletes: A systematic review. Int. J. Sport Nutr. Exerc. Metab..

[25] Trakman G.L., Forsyth A., Devlin B.L., Belski R. (2016). A Systematic Review of Athletes’ and Coaches’ Nutrition Knowledge and Reflections on the Quality of Current Nutrition Knowledge Measures. Nutrients.

[26] Heaney S., O’Connor H., Naughton G., Gifford J. (2008). Towards an understanding of the barriers to good nutrition for elite athletes. Int. J. Sport. Sci. Coach..

[27] Valliant M.W., Emplaincourt H.P., Wenzel R.K., Garner B.H. (2012). Nutrition education by a registered dietitian improves dietary intake and nutrition knowledge of a NCAA female volleyball team. Nutrients.

[28] Hull M.V., Neddo J., Jagim A.R., Oliver J.M., Greenwood M., Jones M.T. (2017). Availability of a sports dietitian may lead to improved performance and recovery of NCAA division I baseball athletes. J. Int. Soc. Sports Nutr..

[29] Jagim A.R., Fields J.B., Magee M., Kerksick C., Luedke J., Erickson J., Jones M.T. (2021). The Influence of Sport Nutrition Knowledge on Body Composition and Perceptions of Dietary Requirements in Collegiate Athletes. Nutrients.

[30] Manore M.M., Patton-Lopez M.M., Meng Y., Wong S.S. (2017). Sport Nutrition Knowledge, Behaviors and Beliefs of High School Soccer Players. Nutrients.

[31] Patton-Lopez M.M., Manore M.M., Branscum A., Meng Y., Wong S.S. (2018). Changes in Sport Nutrition Knowledge, Attitudes/Beliefs and Behaviors Following a Two-Year Sport Nutrition Education and Life-Skills Intervention among High School Soccer Players. Nutrients.

[32] Brozek J., Grande F., Anderson J.T., Keys A. (1963). Densitometric Analysis of Body Composition: Revision of Some Quantitative Assumptions. Ann. N. Y. Acad. Sci..

[33] Trakman G.L., Forsyth A., Hoye R., Belski R. (2018). Development and validation of a brief general and sports nutrition knowledge questionnaire and assessment of athletes’ nutrition knowledge. J. Int. Soc. Sport. Nutr..

[34] Trakman G.L., Brown F., Forsyth A., Belski R. (2019). Modifications to the nutrition for sport knowledge questionnaire (NSQK) and abridged nutrition for sport knowledge questionnaire (ANSKQ). J. Int. Soc. Sport. Nutr..

[35] Evans J.D. (1996). Straightforward Statistics for the Behavioral Sciences.

[36] Ackerman K.E., Holtzman B., Cooper K.M., Flynn E.F., Bruinvels G., Tenforde A.S., Popp K.L., Simpkin A.J., Parziale A.L. (2019). Low energy availability surrogates correlate with health and performance consequences of Relative Energy Deficiency in Sport. Br. J. Sport. Med..

[37] Ackerman K.E., Stellingwerff T., Elliott-Sale K.J., Baltzell A., Cain M., Goucher K., Fleshman L., Mountjoy M.L. (2020). #REDS (Relative Energy Deficiency in Sport): Time for a revolution in sports culture and systems to improve athlete health and performance. Br. J. Sport. Med..

[38] Arthur-Cameselle J., Quatromoni P. (2010). Factors Related to the Onset of Eating Disorders Reported by Female Collegiate Athletes. Sport Psychol..

[39] Arthur-Cameselle J., Sossin K., Quatromoni P. (2017). A qualitative analysis of factors related to eating disorder onset in female collegiate athletes and non-athletes. Eat. Disord..

[40] Bratland-Sanda S., Sundgot-Borgen J. (2013). Eating disorders in athletes: Overview of prevalence, risk factors and recommendations for prevention and treatment. Eur. J. Sport Sci..

[41] Alkazemi D., Zafar T.A., Ebrahim M., Kubow S. (2018). Distorted weight perception correlates with disordered eating attitudes in Kuwaiti college women. Int. J. Eat. Disord..

[42] Ebrahim M., Alkazemi D., Zafar T.A., Kubow S. (2019). Disordered eating attitudes correlate with body dissatisfaction among Kuwaiti male college students. J. Eat. Disord..

[43] Smith A., Emerson D., Winkelmann Z., Potter D., Torres-McGehee T. (2020). Prevalence of Eating Disorder Risk and Body Image Dissatisfaction among ROTC Cadets. Int. J. Environ. Res. Public Health.

[44] Smith A.B., Gay J.L., Monsma E.V., Arent S.M., Sarzynski M.A., Emerson D.M., Torres-McGehee T.M. (2022). Investigation of Eating Disorder Risk and Body Image Dissatisfaction among Female Competitive Cheerleaders. Int. J. Environ. Res. Public Health.

[45] Joy E., Kussman A., Nattiv A. (2016). 2016 update on eating disorders in athletes: A comprehensive narrative review with a focus on clinical assessment and management. Br. J. Sport. Med..

[46] Beals K.A. (2002). Eating behaviors, nutritional status, and menstrual function in elite female adolescent volleyball players. J. Am. Diet. Assoc..

[47] Devlin B.L., Leveritt M.D., Kingsley M., Belski R. (2017). Dietary Intake, Body Composition, and Nutrition Knowledge of Australian Football and Soccer Players: Implications for Sports Nutrition Professionals in Practice. Int. J. Sport Nutr. Exerc. Metab..

[48] Andrews A., Wojcik J.R., Boyd J.M., Bowers C.J. (2016). Sports Nutrition Knowledge among Mid-Major Division I University Student-Athletes. J. Nutr. Metab..

[49] Torres-McGehee T.M., Pritchett K.L., Zippel D., Minton D.M., Cellamare A., Sibilia M. (2012). Sports nutrition knowledge among collegiate athletes, coaches, athletic trainers, and strength and conditioning specialists. J. Athl. Train..

[50] Janiczak A., Devlin B.L., Forsyth A., Trakman G.L. (2022). A systematic review update of athletes’ nutrition knowledge and association with dietary intake. Br. J. Nutr..

